# Supplementary material for: Comparison of the Molecular Motility of Tubulin Dimeric Isoforms: Molecular Dynamics Simulations and Diffracted X-ray Tracking Study
Source: Int J Mol Sci. 2023 Oct 21;24(20):15423. doi: 10.3390/ijms242015423 (PMC10607685; doi:10.3390/ijms242015423)
Supplement: Supplementary file 1 [file ijms-24-15423-s001.zip › ijms-2560132-supplementary.pdf]

Supporting Material

Comparison of the molecular motility of tubulin dimeric isoforms: molecular dynamics simulations and diffracted X-ray tracking study

Tsutomu Yamane, Takahiro Nakayama, Toru Ekimoto, Masao Inoue, Keigo Ikezaki, Hiroshi Sekiguchi, Masahiro Kuramochi, Yasuo Terao, Ken Judai, Minoru Saito, Mitsunori Ikeguchi and Yuji C Sasaki

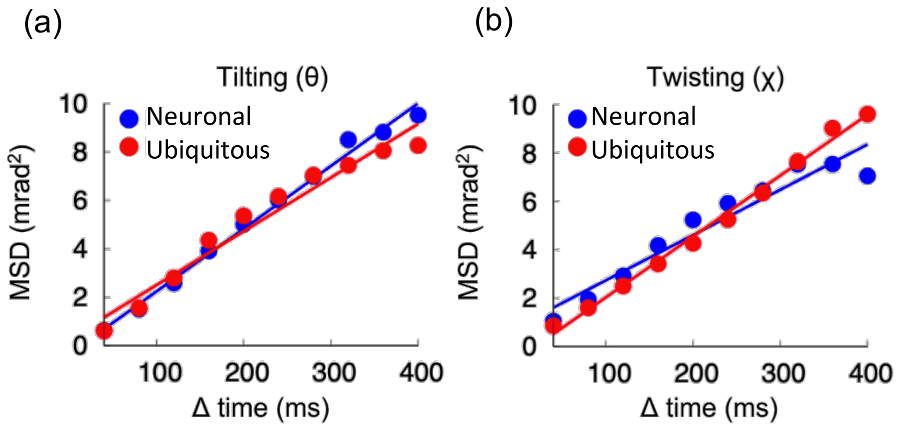

Figure S1. Diffracted X-ray Tracking (DXT) analysis using purified endogenous tubulin dimers.
(a) : Tilting motions of DXT measurement of endogenous tubulin dimers.
(b) : Twisting motions of DXT measurement of endogenous tubulin dimers.
In panels (a) and (b), neuronal and ubiquitous are from brain and each plot have fitting lines drawn by χ-square linear fitting.

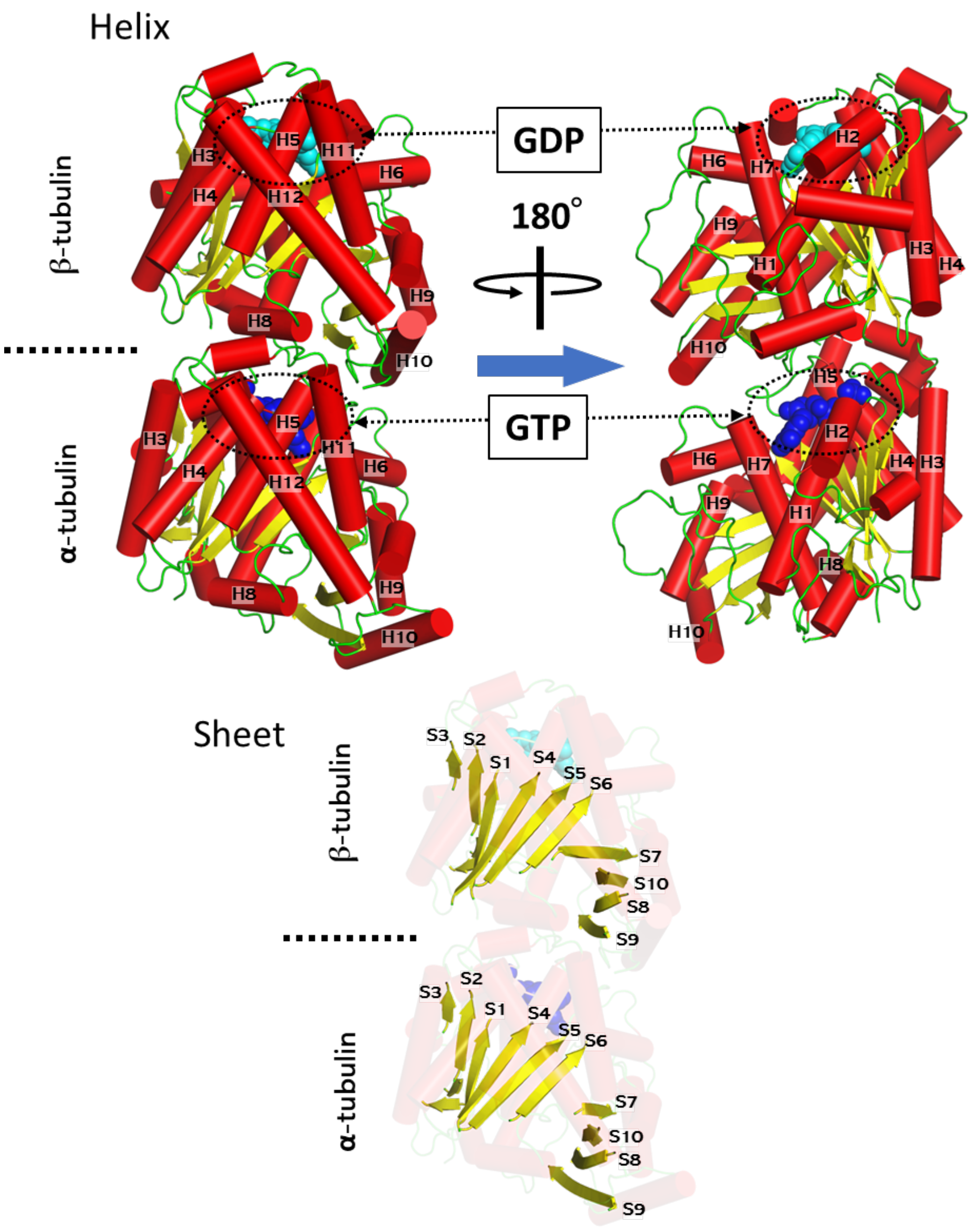

Figure S2. The 3D structural representation of the tubulin dimer (Chains A and B of 3RYC). In the upper panel, the alpha-helices are rendered as red cylinders, with the principal helix names labeled accordingly. The binding guanine nucleotides, GTP and GDP, are highlighted as blue and cyan spheres, respectively. The lower panel distinctively presents the beta-sheets, characterized by yellow arrows and sheet names labeled accordingly. These molecular graphics were drawn by molecular graphics program PyMOL [21].

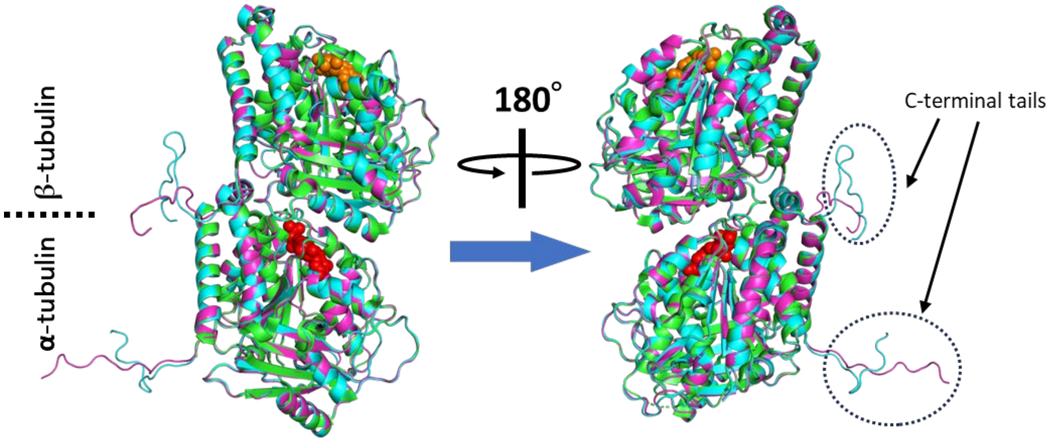

Figure S3. Tubulin dimers modeled in the present study.
In this study, we present the modeled structures of the neuronal tubulin dimer, depicted in cyan, and the ubiquitous tubulin dimer, illustrated in magenta, alongside the template structure used for homology modeling (PDB_ID: 3RYC). The GDP and GTP are represented as spherical models in orange and red, respectively. These molecular graphics were drawn by molecular graphics program PyMOL [21].

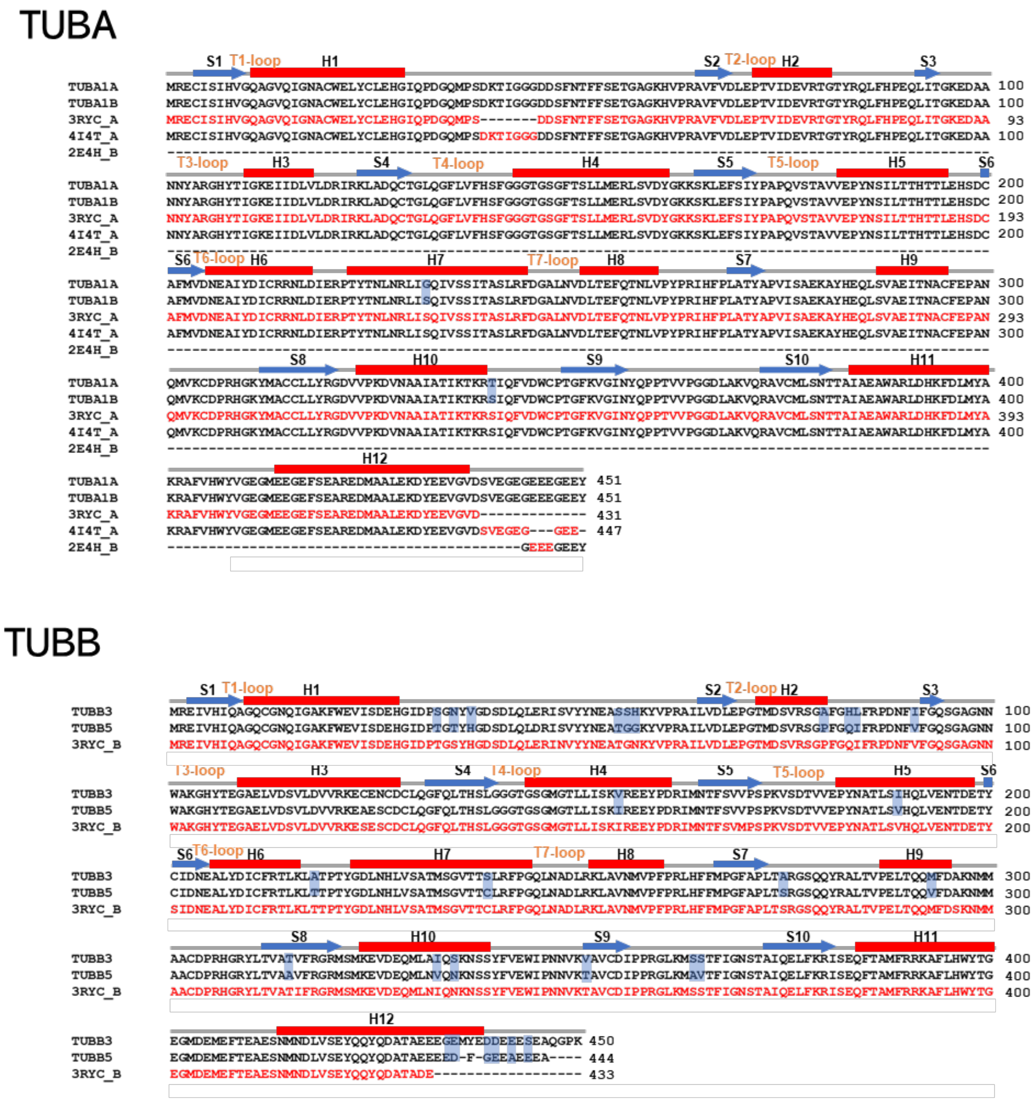

Figure S4. Sequence alignment of PDB structures used as multi-templates in the molecular modeling.

In the provided sequence alignment, the TUBA and TUBB sequences are displayed in the upper and the lower panel, respectively. In these panels, the initial lines represent the tubulin sequences from the brain, followed by their ubiquitous counterparts. Sequences that follow correspond to the PDB templates used in this study. Regions chosen as multi-templates in MODELLER are marked in bold red. For TUBB, the C-terminal tail region beyond Glu433 was modeled autonomously, excluding template structures. Distinct sequence variations between TUBA1A and TUBA1B, and also between TUBB3 and TUBB5, are accentuated with a subtle blue shading. Major secondary structures are depicted in the upper region of sequences: blue arrows for beta-pleats, red squares for alpha-helices, and loops are indicated with orange lettering. These structural distinctions are based on the findings of PDB file of 3RYC and the paper of Lowe [31].

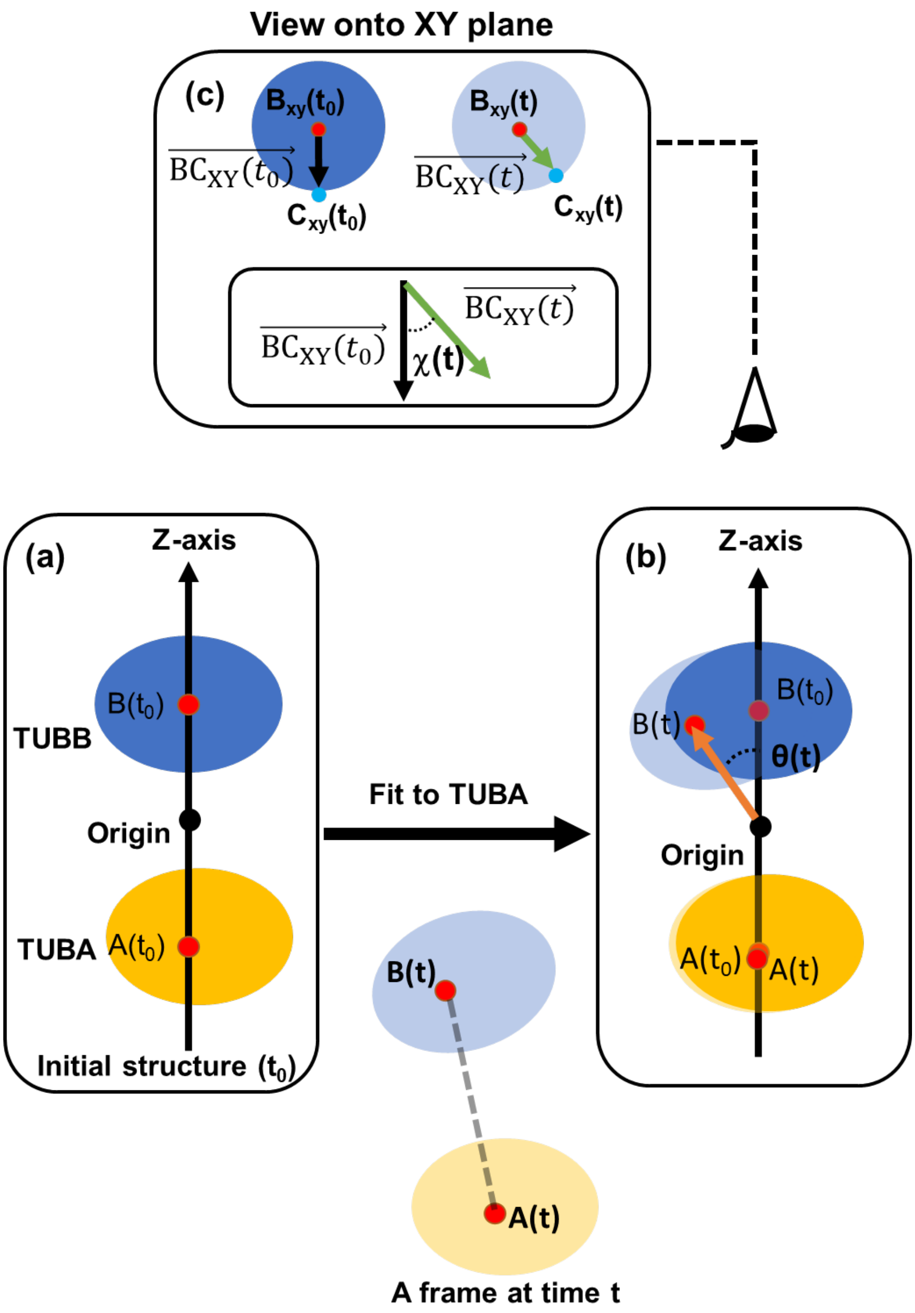

Figure S5. Scheme of analysis of tilt angle and twist angle in MD simulation. The tilt angle θ and twist angle χ were analyzed in the following way.
(a) The centers of mass of TUBA and TUBB in the initial coordinates of the tubulin dimer are A(t_0) and B(t_0), and they are placed on the z-axis, so that the origin is the midpoint of the centers of mass of both dimers.(b) The structure of the trajectory at time t is fitted with TUBA and the angle θ(t) at time t is the angle formed by B(t_0), the origin, and B(t) using the center of mass of TUBB of the trajectory at time t. (c) The center of mass of the part of a beta-sheet (residue numbers 7-9) define C, and define the vectors $\overrightarrow{BC_{XY}(t_0)}$ and $\overrightarrow{BC_{XY}(t)}$, which projected onto the XY plane from the vector $\overrightarrow{BC}$ of the structure at time t_0 and t, which are black and green arrows, respectively. The angle between these two arrows is defined χ(t).

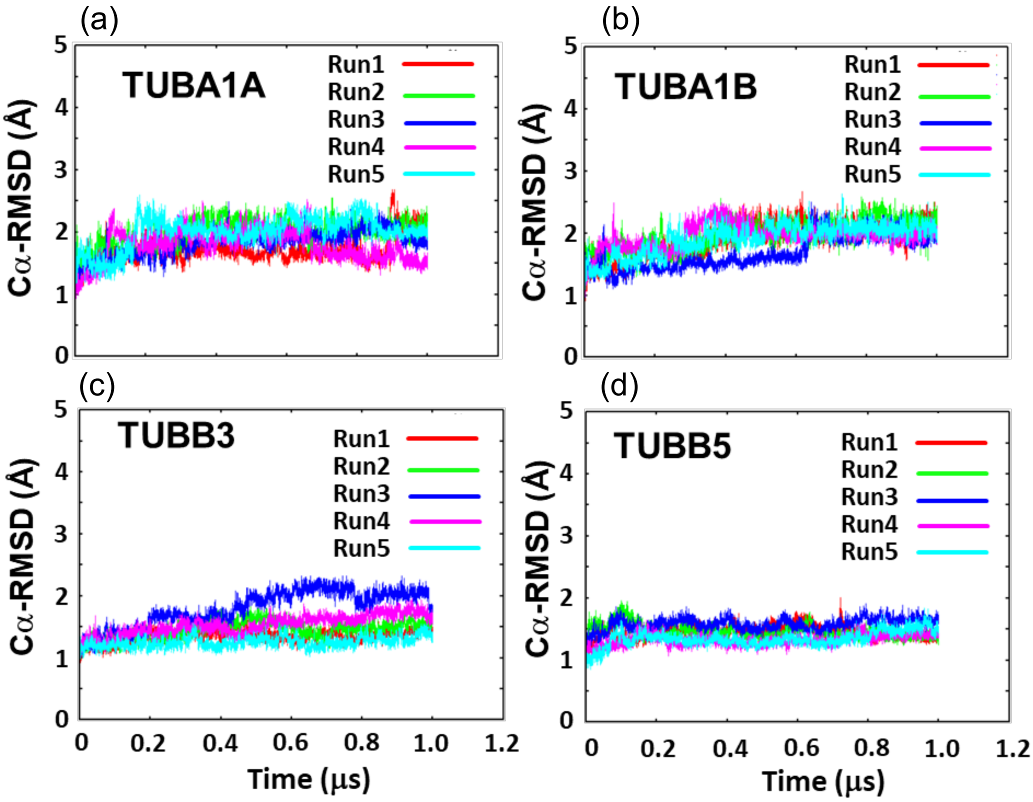

Figure S6. Cα-RMSD of TUBB and TUBA core region.
Cα-RMSDs of the core regions of (a) TUBA1A, (b) TUBA1B, (c) TUBB3, and (d) TUBB5 are shown. The core regions of each tubulin are defined 1-436 (TUBA1A, TUBA1B) and 1-427 (TUBB3, TUBB5). Each panel shows the Cα-RMSD of the five MD simulations.

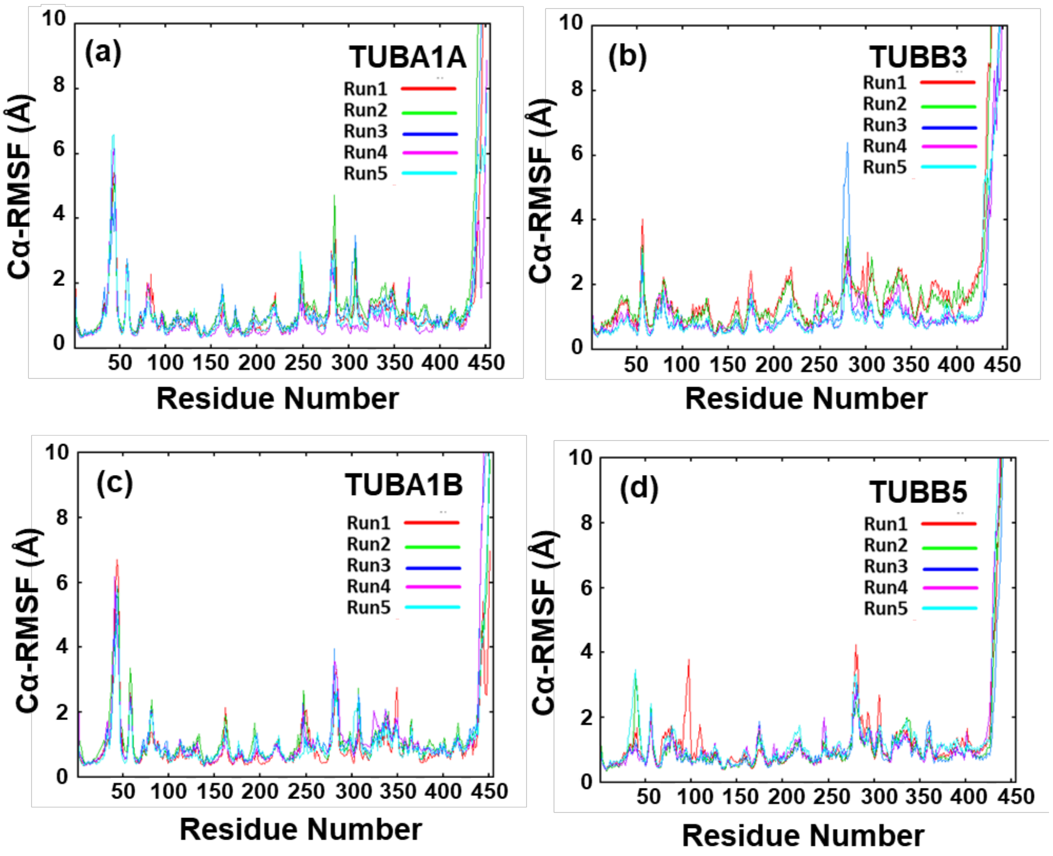

Figure S7. Cα-RMSF (Root Mean Square Fluctuation of Cα atoms) profiles. (a) Cα-RMSF of TUBA1A; (b) Cα-RMSF of TUBB3.; (c) Cα-RMSF of TUBA1B.; (d) Cα-RMSF of TUBB5. The values represent the calculated RMSF based on the results obtained from five independent runs of MD simulations.

Figure SX add

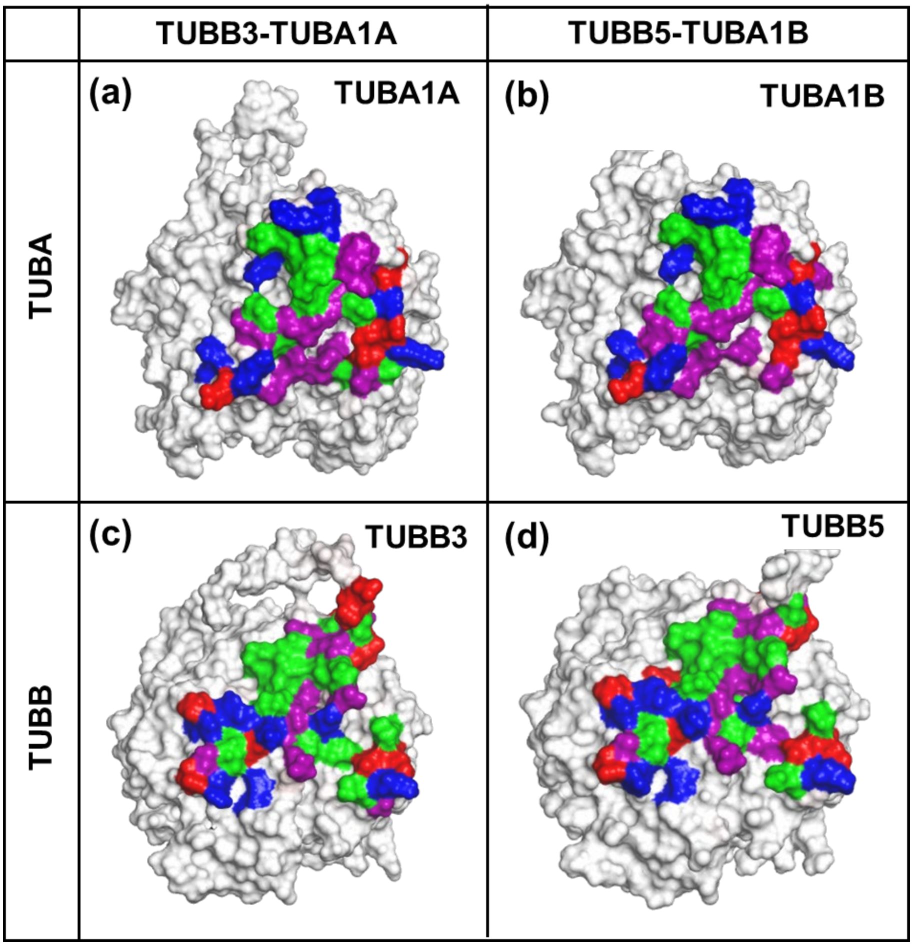

Figure S8. The classifications of amino acid residue at the interface of TUBA and TUBB contact. (a) The interface residues of TUBA1A; (b) The interface residues of TUBA1B; (c) The interface residues of TUBB3; (d) The interface residues of TUBB5. In these panels, the interface amino acids contributed with the contact of TUBA and TUBB, shown in Figure 5, are colored by their characteristics. The colors of residues in these panels are as follows: Hydrophobic amino acids (green): Ala, Val, Phe, Pro, Met, Ile and Leu, Acidic amino acids (red): Asp and Glu, Basic amino acids (Blue): Lys, Arg, Polar amino acids (purple): Ser, Thr, Tyr, His, Cyc, Asn, Gln and Trp. These molecular graphics were drawn by molecular graphics program PyMOL [21].

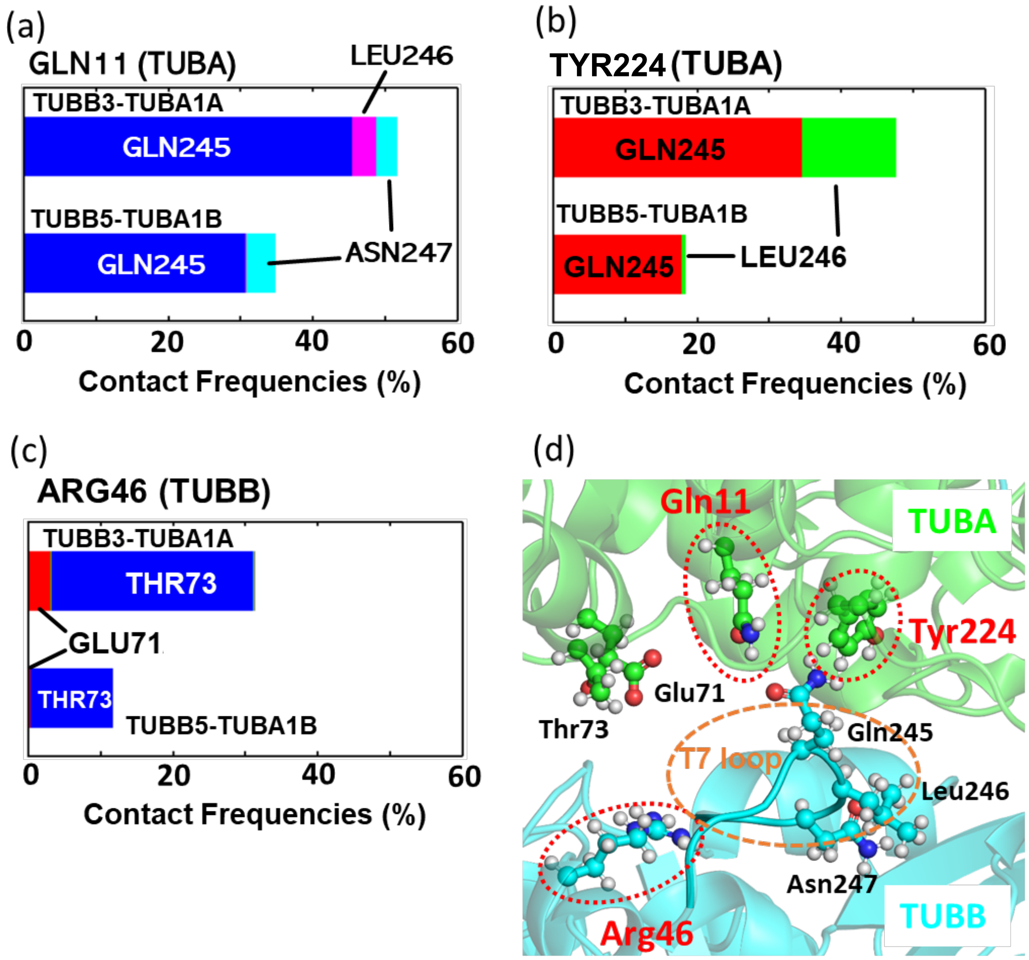

Figure S9. Dimer interactions at the interface.

The contact frequencies and contact partners of the residues (a) Gln11 and (b) Tyr224 in TUBA; and (c) Arg46 in TUBB are shown. In these panels, TUBB3-TUBA1A and TUBB5-TUBA1B are upper and lower bar graph, respectively. The contact frequencies shown in panels (a-c) represent the totals from all MD simulations for TUBB3-TUBA1A and TUBB5-TUBA1B. The standard deviations of the contact frequencies for each amino acid, illustrating the differences among MD runs, are as follows.; GLN11(TUBA): GLN245(TUBB3-TUBA1A, TUBB5-TUBA1B)=(9.8%, 11.4%), LEU246(TUBB3-TUBA1A)=(3.0%), ASN247(TUBB3-TUBA1A, TUBB5-TUBA1B)=(2.7%, 2.2%) TYR224(TUBA): GLN245(TUBB3-TUBA1A, TUBB5-TUBA1B)=(12.3%, 7.2%), LEU246(TUBB3-TUBA1A, TUBB5-TUBA1B)=(11.6%, 0.3%) ARG46(TUBB): GLU71(TUBB3-TUBA1A, TUBB5-TUBA1B)=(6.7%, 0.1%) ,THR73(TUBB3-TUBA1A, TUBB5-TUBA1B)=(1.1%, 3.0%). These contacts were calculated from all of MD simulations and, the residue names of contact partner are shown in the bar graphs in the figure. (d) Residues involved in contacts at the dimer interface. Residues corresponding to (a), (b), and (c) are circled by red dotted lines in the figure. T7 loop is surrounded by an orange dotted line. These molecular graphics were drawn by molecular graphics program PyMOL [21].

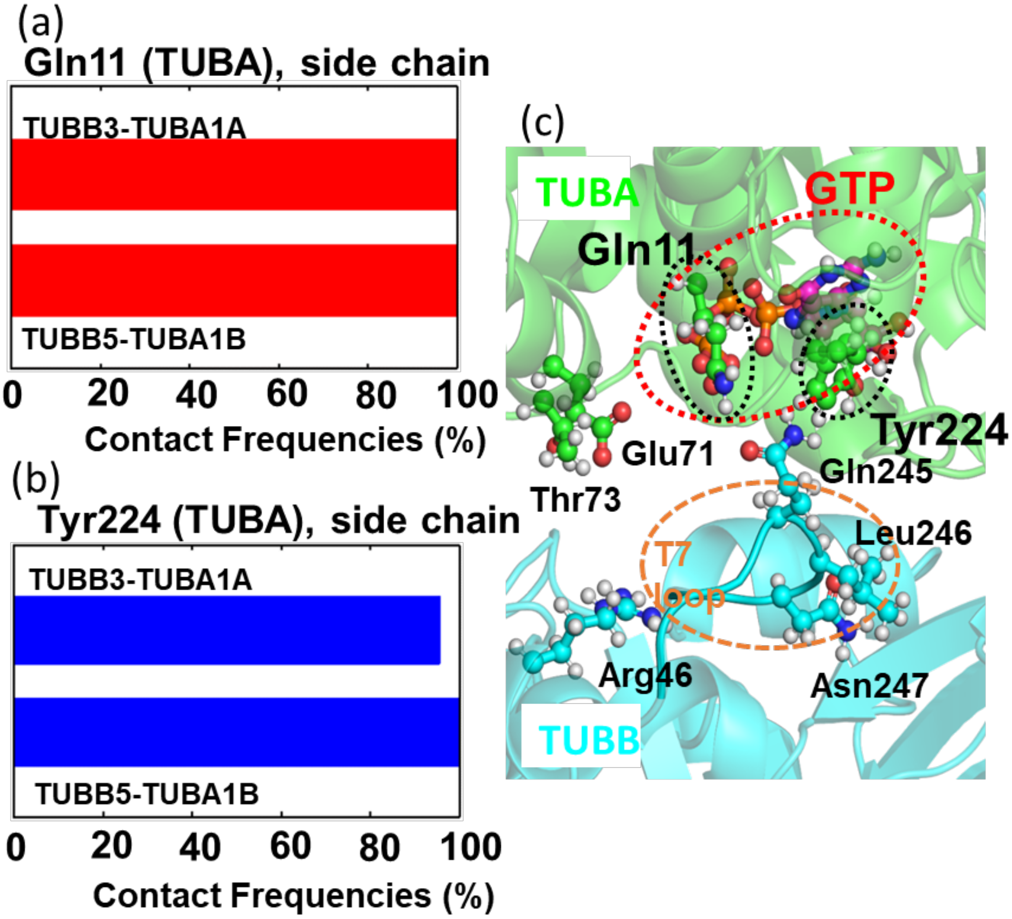

Figure S10. Interaction of GTP with residues of TUBA.
Comparison of TUBB3-TUBA1A and TUBB5-TUBA1B with respect to the frequency of GTP contacts with the side chains of TUBA residues (a) Gln11 and (b) Tyr224. In the figures, the contact frequencies of TUBB3-TUBA1A and TUBB5-TUBA1B are shown in the upper and lower bars, respectively. These figures were computed from trajectories of all MD simulations. The contact frequencies shown in panels (a-b) represent the totals from all MD simulations for TUBB3-TUBA1A and TUBB5-TUBA1B. The standard deviations of the contact frequencies for each amino acid, illustrating the differences among MD runs, are as follows.; GLN11 (TUBA): GTP(TUBB3-TUBA1A, TUBB5-TUBA1B)=(0%, 0%), TYR224(TUBA):GTP(TUBB3-TUBA1A, TUBB5-TUBA1B)=(3.0%, 0%). (c) Diagram of the TUBA and TUBB interface. TUBA and TUBB are shown in green and blue in the figure, and residues involved in interdimer contacts are indicated; GTP is surrounded by a red dotted line, and Gln11 and Tyr224 by black dotted lines. T7 loop is surrounded by an orange dotted line. These molecular graphics were drawn by molecular graphics program PyMOL [21].

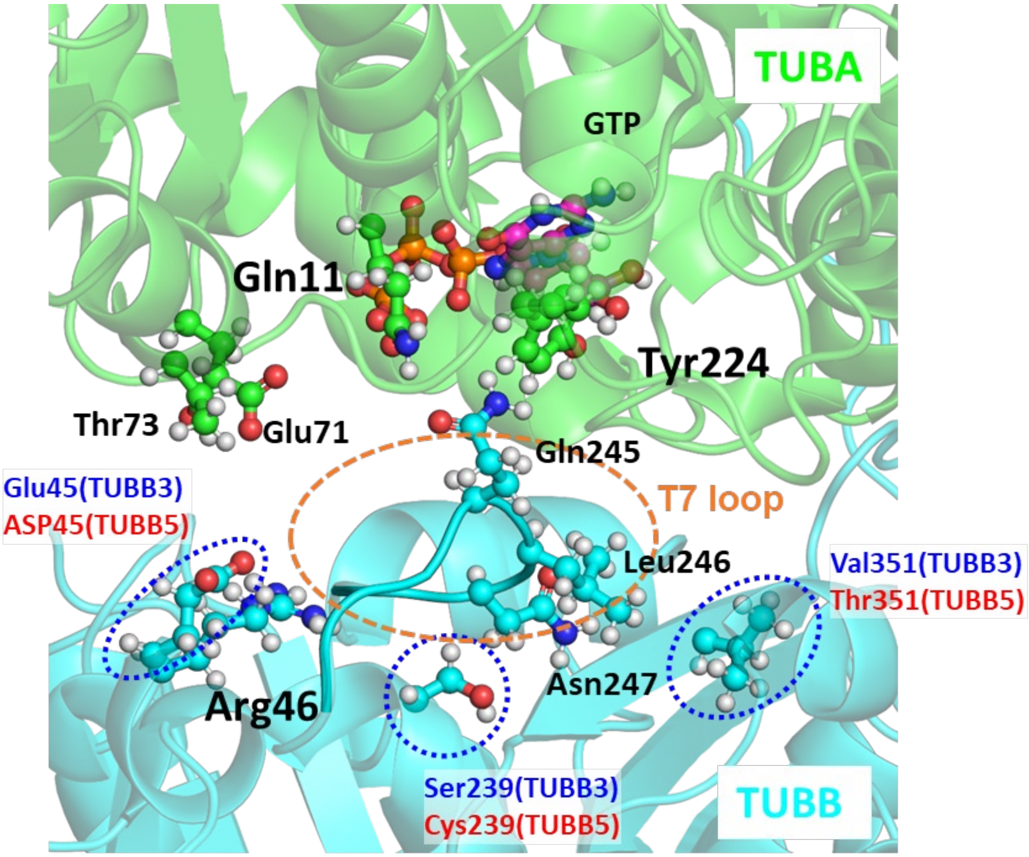

Figure S11. Mutations in the TUBB interface in two tubulin dimers. Mutation of TUBB between TUBB3-TUBA1A and TUBB5-TUBA1B interfaces. In this figure, the structure of TUBB3 is illustrated, and the residues that are mutated with TUBB5 are circled by blue dotted lines. The mutated residues are indicated by red and blue letters for TUBB3 and TUBB5, respectively. Residues involved in major interactions at the interface are also indicated. T7 loop is surrounded by an orange dotted line. These molecular graphics were drawn by molecular graphics program PyMOL [21].

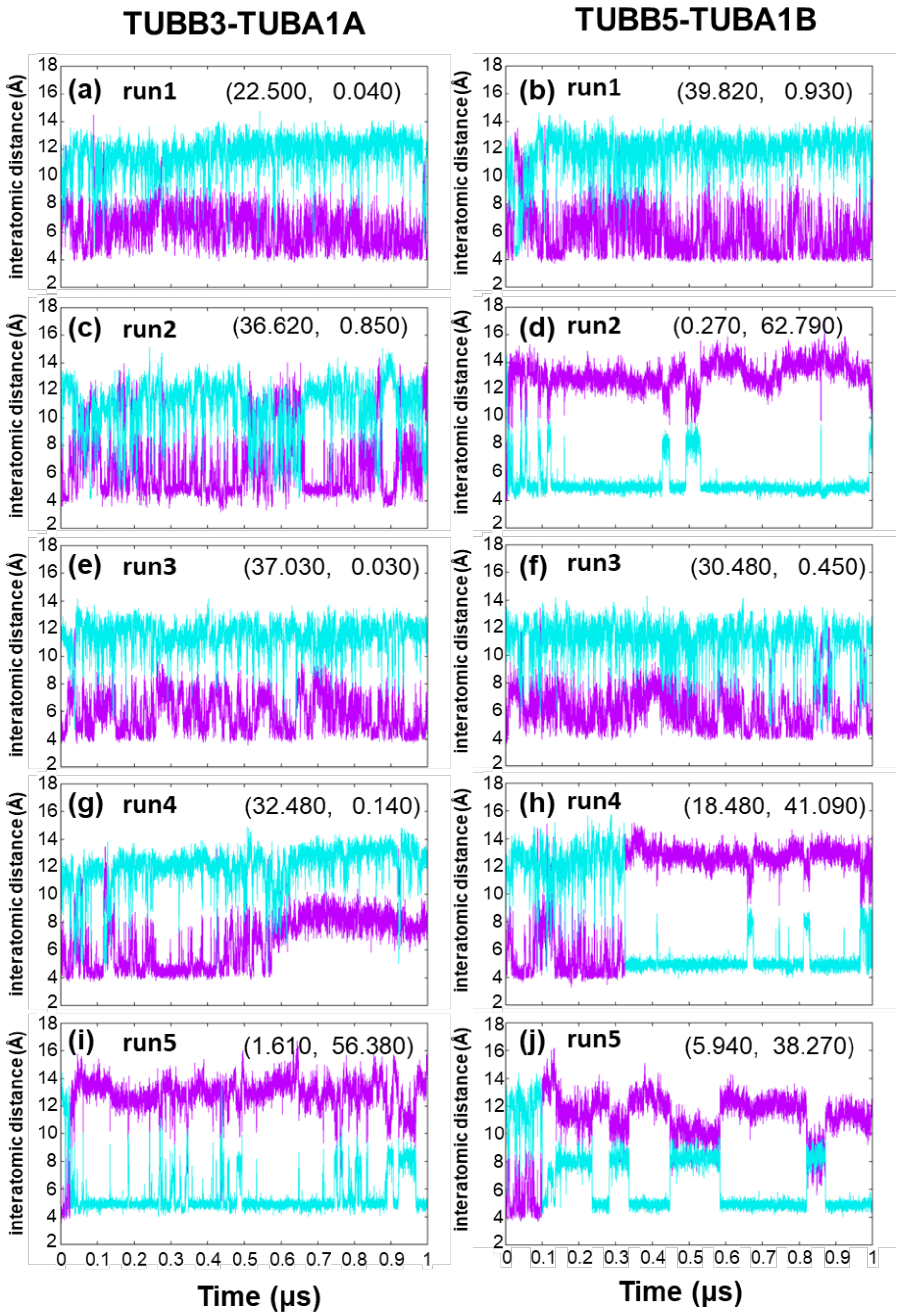

Figure S12. Temporal changes in the interatomic distances between the CD atom of Gln245 and the residues at the TUBB-TUBA interface during MD simulations. These panels depict the temporal changes in interatomic distances during five MD simulations of TUBB3-TUBA1A and TUBB5-TUBA1B: run1 (a, b), run2 (c, d), run3 (e, f), run4 (g, h), and run5 (i, j). In the figures, the interatomic distances CD@Gln245(TUBB)-CD@Gln11(TUBA) and CD@Gln245(TUBB)-CA@Val353(TUBB) are represented by magenta and cyan lines. In the figure, frames where the interatomic distances CD@Gln245(TUBB)-CD@Gln11(TUBA) and CD@Gln245(TUBB)-CA@Val353(TUBB) are less than 5Å are defined as State A and State B, respectively. The percentage of these states in each MD trajectory is indicated in each plot as (State A, State B). respectively

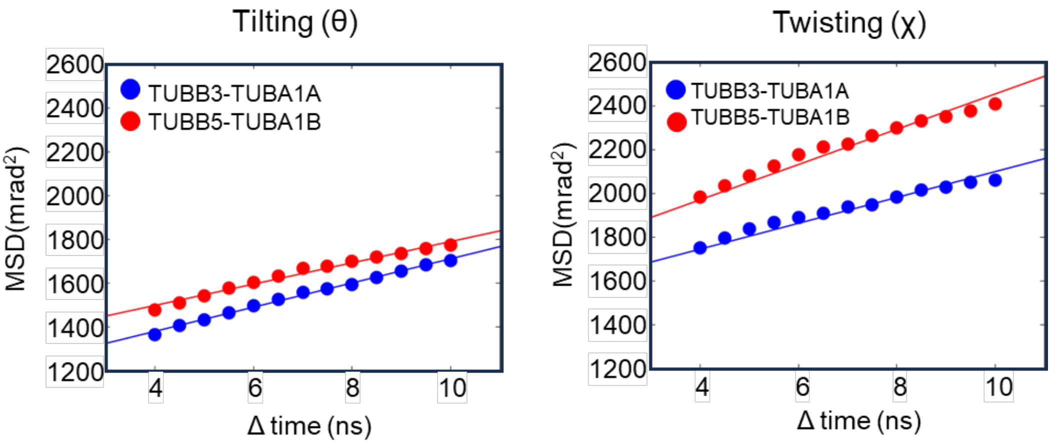

Figure S13. Plots of the time dependence of the MSD of the Tilting (θ) and Twisting (χ) movements obtained from MD simulations. (a) Plot of the time dependence of the MSD of the Tilting motion.(b) Plot of the time dependence of the MSD of the Twisting motionIn these figures, the plots of TUBB3-TUBA1A and TUBB5-TUBA1B are indicated by red and blue dots and lines, respectively. The method shown in Figure S3 was used to calculate these angles. These plots were obtained as the average of five 1 μs MD runs for each of TUBB3-TUBA1A and TUBB5-TUBA1B. The plot of MSD versus time obtained from the MD simulation was calculated using the following method, first, squared displacements between time width t_x, $\left(X(t_x) - X(t_0)\right)^2$ calculated over the trajectory. Where, X is the angle of θ and χ . Then, using the value of the squared displacement at t_x obtained from the trajectory, the value of the MSD was calculated using the following equation:

$$\left\langle \left(X(t_x) - X(t_0)\right)^2 \right\rangle = 1/n \sum_{l=1}^{n} \left(X^l(t_x) - X^l(t_0)\right)^2$$

where n is the number of samples at time width t_x observed in the trajectory.

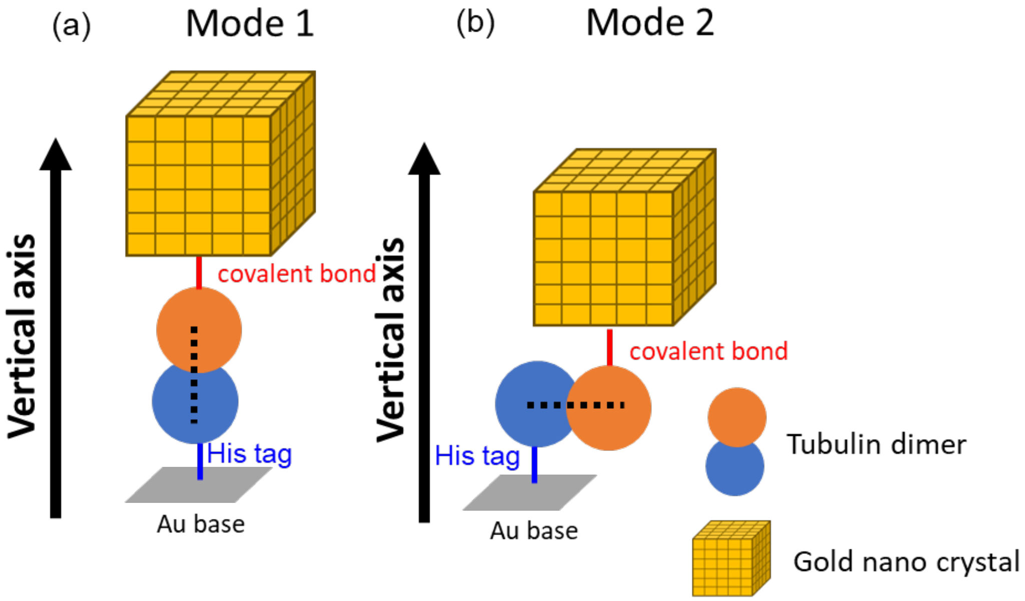

Figure S14. Two types of Modes of orientation of tubulin dimer assumed in DXT experiments.

(a) The orientation mode in which the line connecting tubulin monomers is parallel to the Vertical axis (Mode 1); (b) Orientation in which the line connecting tubulin monomers is perpendicular to the vertical axis (Mode 2). In these panels, the axis connecting the monomers is shown as a dotted line.

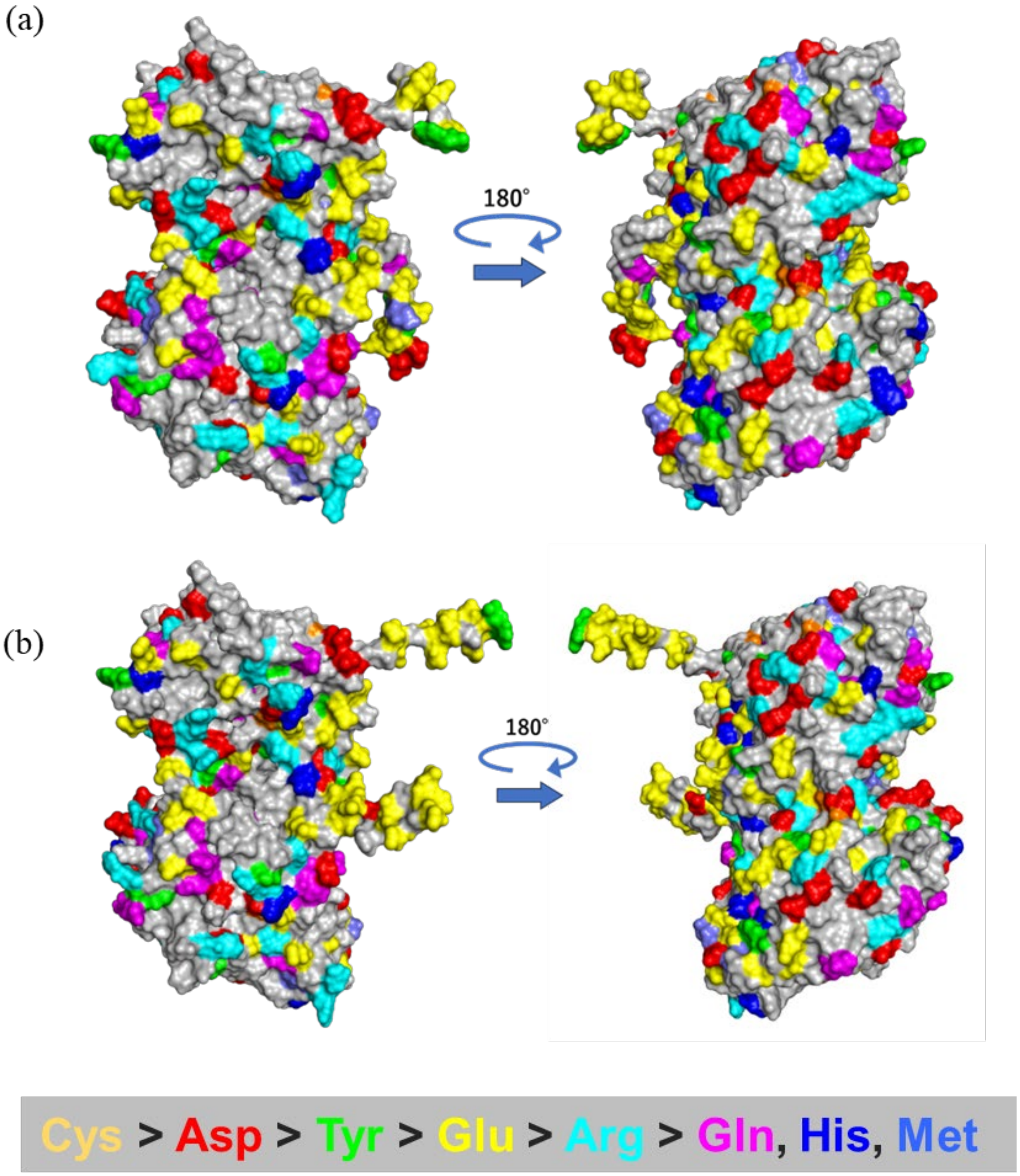

Figure S15. Distribution of amino acids with high binding affinity for gold in tubulin dimers.
(a) Distribution of amino acids with high binding affinity for gold on the molecular surface in TUBB3-TUBA1A. (b) Distribution of amino acids with high binding affinity for gold on the molecular surface in TUBB5-TUBA1B. The structures shown in these panels were modeled in this study. Panels (a) and (a) show the Accessible Surface Area of the tubulin dimer. These molecular graphics were drawn by Pymol [46]. The colors of each amino acid in the figure are as follows: cysteine (orange), aspartate (red), tyrosine (green), glutamic acid (yellow), arginine (cyan), glutamine (magenta), histidine (blue), methionine (pale blue). These figures are drawn by molecular graphics program PyMOL [21].

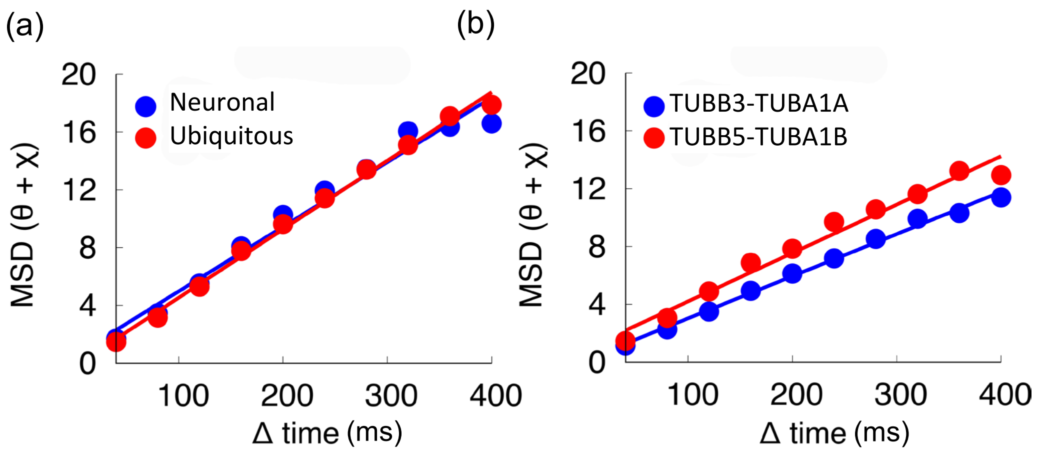

Figure S16. MSD results for the sum of Tilting(θ) and Twisting(χ) motions obtained by X-ray diffraction tracking (DXT) analysis.

(a) : The results of MSD of θ+χ obtained from DXT analysis of purified endogenous tubulin dimers.

(b) : The results of MSD of θ+χ obtained from DXT analysis of tubulin dimers reconstructed from purified recombinant subunit proteins.

In panel (a), the plots of the Neuronal and Ubiquitous are colored blue and red, respectively.

In panel (b), the plots of the main components of Neuronal (TUBB3-TUBA1A) and Ubiquitous (TUBB5-TUBA1B) are colored blue and red, respectively.

Table S1. Summary of diffusion constants of TUBB3-TUBA1A and TUBB5-TUBA1B obtained from DXT experiments of recombinant protein and MD simulations.

		Diffusion constant ($mrad^2/ms$)	
		TUBB3-TUBA1A*** (neuronal)	TUBB5-TUBA1B*** (ubiquitous)
Tilting (θ)	DXT*	0.00450578 +/- 0.0001086	0.00529076 +/- 0.0002487
	MD**	0.009206 +/- 0.000182 ($x10^9$)	0.008103 +/- 0.000323 ($x10^9$)
Twisting (χ)	DXT*	0.00278531 +/- 0.000135	0.00307847 +/- 0.0002674
	MD**	0.009830 +/- 0.000418 ($x10^9$)	0.013421 +/- 0.000577 ($x10^9$)

*The diffusion constants D were obtained from chi-square liner fitting with y=4Dt+b based on MSD plots (Figure 1c, d).

**The diffusion constants D were obtained from chi-square liner fitting with y=6Dt+b based on MSD plots (Figure S8a, b).

***TUBB3-TUBA1A and TUBB5-TUBA1B are main components of tubulin dimer of neuronal and ubiquitous, respectively.

Table S2. Summary of diffusion constants of native tubulin dimer obtained from DXT experiments of purified endogenous tubulin dimers.

		Diffusion constant (mrad^2/ms)	
		Neuronal	Ubiquitous
Tilting (θ)	DXT*	0.00648395 +/- 0.0002077	0.00555622 +/- 0.0004008
Twisting (χ)	DXT*	0.00468853 +/- 0.000462	0.00630033 +/- 0.0001829

*The diffusion constants D were obtained from chi-square liner fitting with y=4Dt+b based on MSD plots (Figure S1a and S1b).

Table S3. Summary of diffusion constants for the sum of tilting and twisting motions from DXT experiments on endogenous and recombinant tubulin dimers.

	Diffusion constant ($mrad^2/ms$) *	
	Neuronal** (TUBB3-TUBA1A)	Ubiquitous** (TUBB5-TUBA1B)
Endogenous	0.0111725 +/- 0.0006528	0.0118565 +/- 0.0003024
Reconbinant	0.0072911 +/- 0.0001956	0.00836924 +/- 0.0004797

*The diffusion constants D were obtained from chi-square liner fitting with y=4Dt+b based on MSD plots (Figure S11a and S11b).

**Parentheses indicate recombinant tubulin dimers.
